# First isolation and genomic characterization of enterovirus A71 and coxsackievirus A16 from hand foot and mouth disease patients in the Lao PDR

**DOI:** 10.1002/nmi2.63

**Published:** 2014-09-18

**Authors:** V H Nguyen, B Sibounheuang, K Phommasone, M Vongsouvath, P N Newton, G Piorkowski, C Baronti, X de Lamballerie, A Dubot-Pérès

**Affiliations:** 1 Aix Marseille Université, IRD French Institute of Research for Development, EHESP French School of Public Health; 2 IHU Méditerranée Infection, APHM Public Hospitals of MarseilleMarseille, France; 3 Lao-Oxford-Mahosot Hospital-Wellcome Trust Research Unit (LOMWRU), Microbiology Laboratory, Mahosot HospitalVientiane, Lao PDR; 4 Centre for Tropical Medicine, Nuffield Department of Medicine, Churchill Hospital, University of OxfordOxford, United Kingdom

**Keywords:** Coxsackievirus A16, enterovirus A71, genome sequence, hand, foot and mouth disease

## Abstract

Enterovirus A71 (EV-A71) and coxsackievirus A16 (CV-A16) are major aetiological agents of hand, foot and mouth disease in Asia. We established the first genomic characterization of strains isolated in 2011 from Lao patients. Isolates were related to EV-A71 genotype C4 and CV-A16 genotype B1a that circulated in neighbouring countries during the same period. This confirms the regional character of hand, foot and mouth disease epidemiology and makes plausible the occurrence of severe disease in the Lao population.

## Introduction

Hand, foot, and mouth disease (HFMD) is a common childhood disease, most often characterized by fever, pharyngitis, mouth ulcers and papulo-vesicular rash on the palms and soles. Over the last decade HFMD has emerged as a growing public health issue in Asia with an increased burden of severe disease 1–4. The illness can be due to numerous members of the *Enterovirus* genus (family *Picornaviridae*, order *Picornavirales*). Coxsackievirus A16 (CV-A16) and enterovirus A71 (EV-A71) (species *Enterovirus A*), are major aetiological agents in Asia and often co-circulate during outbreaks 5. The illness caused by CV-A16 infection is usually mild, whereas infections by EV-A71 have been associated with significant morbidity and mortality, particularly in the Asia-Pacific region. Severe complications include brainstem encephalitis, aseptic meningitis, severe pulmonary oedema and cardio-respiratory collapse 6,7. Enteroviruses (EV) are non-enveloped viruses transmitted by direct contact with saliva, faeces, vesicular fluid or respiratory droplets. The viral genome is a single-stranded positive-sense RNA (˜ 7.5 kb) with a unique open reading frame flanked by two untranslated regions (5′ and 3′ UTRs). The polyprotein is cleaved in four capsid proteins, VP1–4, and seven non-structural proteins, 2A–C, 3A–D. h VP1 sequences has become the method of reference for EV typing 8. EV-A71 have been initially classified into three genotypes, A, B (subgenotypes B0–B5) and C (subgenotypes C1–C5), but three additional genotypes (D–F) have recently been described 9. CV-A16 are divided in three genotypes A, B1 (subgenotypes a–c), and B2 10.

There are no published data on the epidemiology of HFMD in the Lao PDR and EV-A71 cases have not been reported, although paediatricians are concerned that they have seen HFMD patients. Importantly, and in contrast with neighbouring countries, no patients with severe disease have been reported to date in Laos. This may be due do the lack of available diagnostic tests or to specific epidemiological characteristics, but also raises the issue of the possible circulation of specific low-pathogenic viral variants. To examine the latter issue, samples from children presenting at hospital with symptoms and signs of HFMD have been collected at Mahosot Hospital, Vientiane, more than 200 since June 2010, and analysed for EV detection and characterization.

EV-A71 and CV-A16 were isolated from two HFMD patients, HFMD12 and HFMD4, attending Mahosot Hospital in February and May 2011, respectively. Both children, aged 15 and 18 months old respectively, presented with typical mouth ulcers and vesicles on hands, feet, trunk and buttocks. Throat and vesicle swabbing was performed using Sigma Virocult® (MWE, Corsham, UK) for both children and blood was collected only for HFMD12.

Two hundred microlitres of transport medium or serum was extracted using the EZ1 Virus Mini-Kit v2.0 (Qiagen, Hilden, Germany) and enteroviral genomes were subsequently detected using a Pan-EV real-time RT-PCR system as described previously 11.

One hundred microlitres of transport medium from vesicle swab was inoculated on MA104 cells and culture medium was collected 4 days after inoculation when gross cytopathic effect was observed. RNA was extracted from 200 μL as reported above. EV were typed by VP1 amplification and sequencing according to Nix *et al*. 12. Genomic amplification was performed using random (CV-A16) or specific (EV-A71) protocols followed by Next-Generation Sequencing using the Ion-Torrent Personal Genome Machine (Life Technologies, Carlsbad, CA, USA).

The almost complete genome sequences of EVA71/LA/HFMD12V/2011 (GenBank KM055005); 7379 nucleotides long including the full open reading frame, 723 nucleotides of 5′ UTR and 77 nucleotides of 3′ UTR) and of CVA16/LA/HFMD4V/2011 (GenBank KM055004); 6971 nucleotides long including the full open reading frame, 288 nucleotides of 5′ UTR and 104 nucleotides of 3′ UTR) were obtained. Both viruses were deposited in the European Virus Archive (EVA) under the references 977 and 1013, respectively.

**Figure 1:**
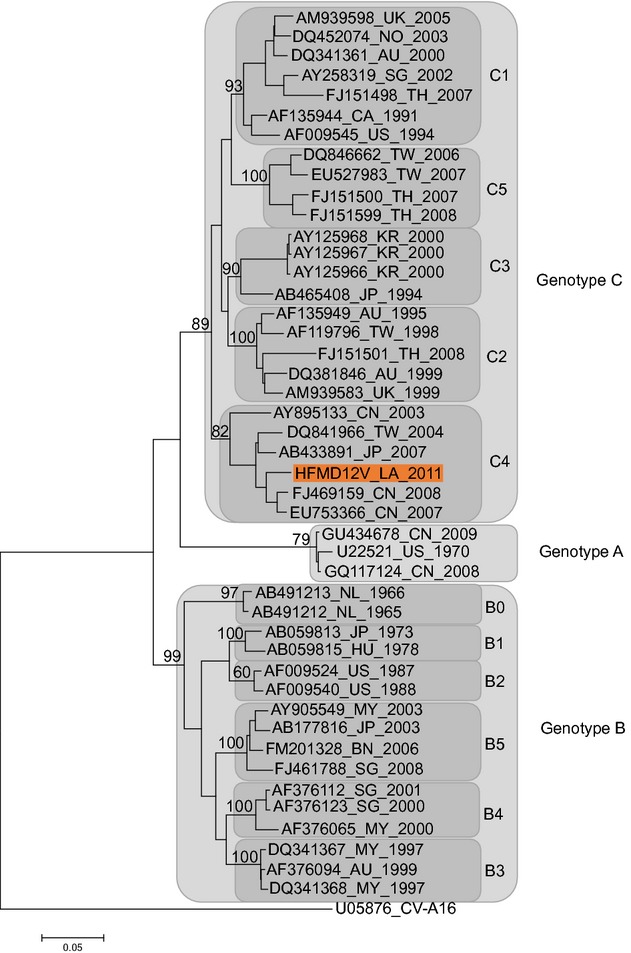
Neighbour-joining tree of enterovirus A71 (EV-A71) VP1 sequences. Tree produced using mega 6.06 software with Kimura-2 distance calculation algorithm with VP1 sequences from 44 EV-A71 representatives of the genotypes A, B, and C aligned using clustalx 2.1. Bootstrap values (in percentage) were generated by using 500 replicates. For each strain, the GenBank accession number, the country of origin (ISO 3166 code) and the year are indicated, excepted for the strain isolated in Lao PDR in 2011, which is highlighted in red.

**Figure 2:**
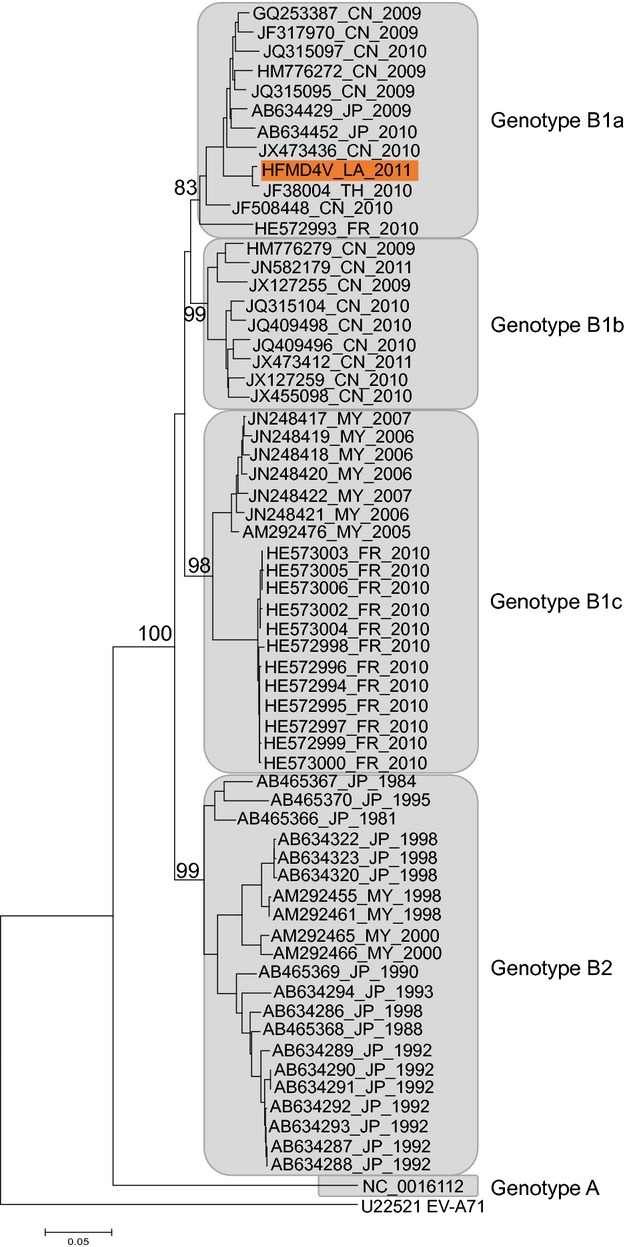
Neighbour-joining tree of coxsackievirus A16 (CV-A16) VP1 sequences. Tree produced using mega 6.06 software with Kimura-2 model with VP1 sequences from 61 CV-A16 representatives of the genotypes B1a, B1b, B1c and B2, and the prototype BC001612 of genotype A, aligned using clustalx 2.1. Bootstrap values (in percentage) were generated by using 500 replicates. For each strain, the GenBank accession number, the country of origin (ISO 3166 code) and the year are indicated, excepted for the strain isolated in Lao PDR in 2011, which is highlighted in red.

The VP1 sequence of HFMD12V was aligned with 44 other EV-A71 sequences from genotypes A–C, and HFMD4V was aligned with 61 CV-A16 sequences using clustalX2.1 13. Neighbour-joining trees were constructed using mega 6.06 with the Kimura-2 distance algorithm 14 and bootstrap resampling with 500 replicates. The EV-A71 tree (Fig.1) revealed that the HFMD12V strain belongs to genotype C4, which is the predominant genotype circulating in the Asia-Pacific region 15. BLAST nucleotide analysis identified 98% identity with strains HQ456308 and HQ712020 isolated in China in 2010, in Guangdon and Beijing, respectively. The CV-A16 tree (Fig.2) showed that strain HFMD4V belongs to genotype B1a, with 98% and 96% identity with a strain isolated in Thailand in 2010 (GenBank JF738004) and a strain from Yunnan, China 2008 (GenBank HQ423141), respectively.

This study demonstrates the circulation in Laos of EV-A71 genotype C4, which is associated elsewhere in South-East Asia with severe disease. This has important public health implications, suggesting that the Lao population is exposed to the possible occurrence of severe HFMD, as observed in neighbouring countries.

## Conflict of Interest

None declared.
